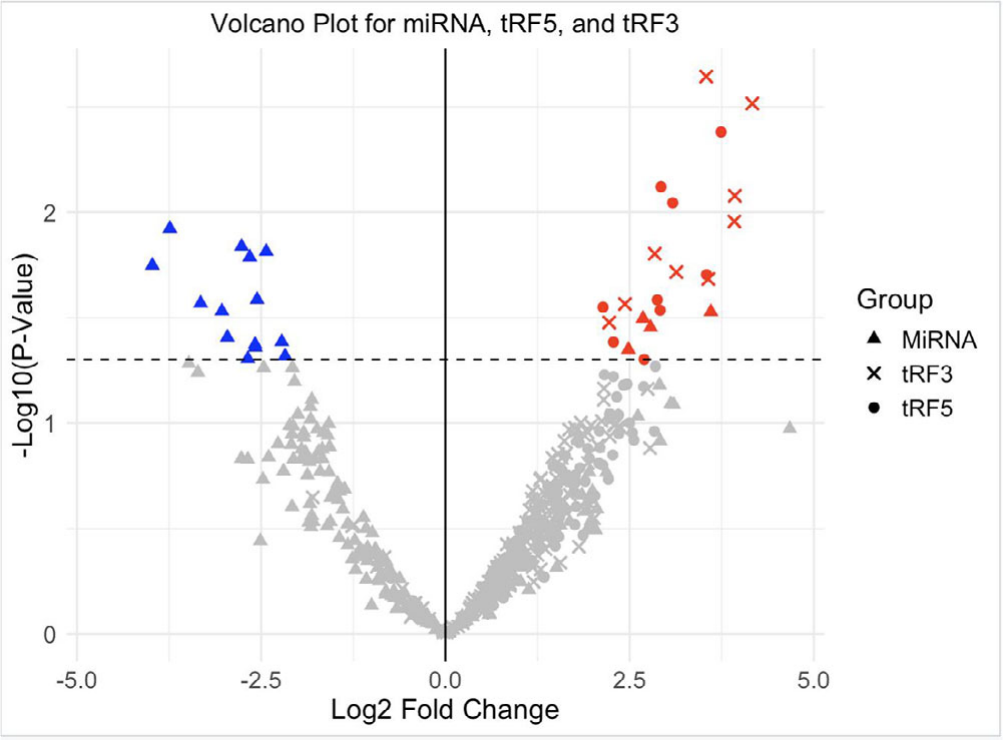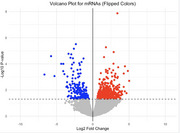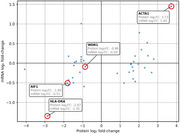# Negatively‐correlated tRF5 expressions with mRNA and protein expressions in iPSC‐derived microglia cells in Alzheimer’s disease

**DOI:** 10.1002/alz70861_108932

**Published:** 2025-12-23

**Authors:** Veena Thamilselvan, Luke Liu, Jared Lin, Wenzhe Wu, Eun Seok Choi, Xiaoyong Bao, Inhan Lee

**Affiliations:** ^1^ miRcore, Ann Arbor, MI USA; ^2^ University of Texas Medical Branch, Galveston, TX USA

## Abstract

**Background:**

Studies have identified neurotoxic microglial activity in the progression of Alzheimer’s disease (AD). tRNA‐derived fragments (tRFs) have been implicated in transcription/translation modulation. Understanding how specific tRFs regulate microglial gene expression could provide insights into molecular crosstalk underlying AD and disease progression.

**Method:**

3 control and 3 Alzheimer iPSCs patient samples from the Coriell Institute were first differentiated into hematopoietic progenitor cells, and then into microglia cells. Small RNAs (sRNA), total RNAs, and proteins were extracted from the microglia cells and their expressions measured with RNAseq and MALDI‐TOF.

Our sRNA database contained miRNA, tRFs, snRNAs, snoRNAs, and piRNAs; a mRNA database was prepared using RefSeq transcripts. Bowtie2 was used to map reads and DESeq2 to analyze differential genes. For target prediction, we used both interaction energies between tRF5 and UTRs (RNAhybrid) as well as consecutive matches of 8‐mer or more.

**Result:**

After the DESeq2 analysis of sRNAs and mRNAs, volcano plots of miRNAs, tRF5, and tRF3 among sRNAs (Figure 1) and of mRNA (Figure 2) were compared. While miRNAs were more downregulated, all significant tRF5s and tRF3s were upregulated (p < 0.05). Figure 3 shows a comparison between mRNA expression and proteomics data. Among them, ACTA1 is most upregulated in both protein and mRNA while HLA‐DRA is among the most strongly downregulated. To identify their potential targets, here we focus on tRF5s since they are all upregulated and more abundantly expressed than tRF3s. Interestingly, all downregulated genes, in terms of both mRNA and protein levels, were negatively correlated with certain tRF5s and had strong interaction sites with their UTRs. Further, multiple upregulated tRF5s had strong interaction sites with specific genes, including four out of nine upregulated tRF5s with HLA‐DRA as well as seven tRFs with WDR1.

**Conclusion:**

Analysis of iPSC‐derived microglia revealed significant in vitro tRF5 expression differences and related mRNA/protein targets. HLA‐DR and WDR1 proteins were identified as downregulated and negatively correlated with multiple tRFs (correlation *p* < 0.05) while their respective mRNA UTRs and tRFs had strong interaction sites, suggesting tRF5 regulation with these genes. We also noted downregulated HLA‐DR and AIF1, suggesting roles of brain microenvironments in disease pathology.